# Venous Excess Ultrasound (VExUS) score and short-term outcomes in ambulatory patients with heart failure with reduced ejection fraction: an exploratory study

**DOI:** 10.1186/s43044-025-00648-w

**Published:** 2025-06-13

**Authors:** Ahmed Hassan, Ahmed A. Khalil, Amir Mostafa, Hesham Yehia

**Affiliations:** Cardiology Department, cairo university, Cairo, Egypt

**Keywords:** Congestion, Heart failure, Hospitalization, VExUS score, Tricuspid regurgitation, Inferior vena cava diameter

## Abstract

**Background:**

Venous congestion significantly contributes to morbidity and mortality in heart failure with reduced ejection fraction (HFrEF). The Venous Excess Ultrasound (VExUS) score, a multi-parameter sonographic assessment of systemic venous congestion, has shown prognostic utility in hospitalized HFrEF. However, its role in risk-stratifying ambulatory HFrEF patients remains unclear. This prospective exploratory study investigated the prognostic value of elevated VExUS scores in ambulatory HFrEF patients.

**Results:**

Of 109 enrolled patients, 23 (21%) had a high VExUS score (≥ 2). Patients with high VExUS scores had significantly higher rates of the primary composite endpoint of all-cause mortality or HF hospitalization within 90 days (87% vs. 19.8%, *p* < 0.001), worsening renal function (47.8% vs. 17.3%, *p* < 0.001), and unplanned visits (82.6% vs. 14.8%, *p* < 0.001). While dilated IVC had higher sensitivity for the primary outcome, VExUS demonstrated greater specificity, especially in patients with moderate to severe tricuspid regurgitation (TR).

**Conclusions:**

An elevated VExUS score was associated with adverse short-term outcomes in this exploratory analysis. VExUS score may provide a more specific assessment of congestion than IVC diameter alone, particularly in the presence of significant TR, potentially improving risk stratification in this population.

## Background

Heart failure (HF) poses a growing global health burden, placing substantial strain on healthcare resources. Acute decompensated heart failure (ADHF), marked by a rapid or gradual symptom exacerbation requiring urgent intervention, frequently leads to unscheduled hospitalizations and emergency department visits [1]. ADHF is a leading cause of hospitalization in adults over 65, associated with considerable in-hospital mortality (4–10%) and high readmission rates [2, 3]. Rehospitalization rates for HF patients can reach 20% within 30 days and 50% within six months post-discharge [4], with some studies reporting rates as high as 55% within three months.

While various clinical and laboratory indicators, such as clinical assessment of congestion, serum sodium, and B-type natriuretic peptide, are used to predict readmission and mortality [4, 5], accurately evaluating a patient's congestive state remains challenging. Traditional methods, including jugular venous pressure assessment and evaluation for peripheral edema and rales, are often insufficient for detecting subclinical or early hemodynamic congestion crucial for prognostication [5]. While useful in advanced HF, these clinical findings lack sensitivity for early volume overload [6] and are subject to inter-observer variability [7].

Ultrasonographic parameters offer improved sensitivity for detecting early congestion and complement traditional assessments [8, 9]. The Venous Excess Ultrasound (VExUS) score, a novel multi-parameter ultrasound assessment of systemic venous congestion developed by Beaubien-Souligny et al. [10], has shown a good prognostic value in post-cardiac surgery acute kidney injury (AKI). The VExUS score evaluates abnormalities in the inferior vena cava (IVC), hepatic, portal, and renal veins, reflecting the retrograde progression of venous congestion from the IVC [10]. Its correlation with right atrial pressure (RAP) [11] suggests utility in HF management.

Data on the VExUS score in heart failure are limited; only the individual parameters of the VExUS score, not the full scoring system, have been studied in both hospitalized and outpatient HF patients [12–14].

The objective measure of congestion holds promise for refining HF therapies. Lung ultrasound, for instance, has shown predictive value for hospitalization and mortality risk in several studies of ambulatory heart failure patients and can guide diuretic optimization [15]. Likewise, assessment of the IVC demonstrates an 82% sensitivity for predicting elevated RAP (> 10 mmHg), significantly outperforming clinical jugular venous pulse inspection [16], and it became a readily available and prognostically valuable tool for assessing systemic congestion. However, it is a subject of some confounders like significant tricuspid regurgitation, COPD exacerbation, and elevated intra-abdominal pressure. [9] The portal vein has been shown to effectively guide decongestion in ADHF patients with AKI when IVC assessment is inadequate [17]. Renal vein Doppler waveforms have also demonstrated prognostic significance [17].

Integrating these advanced ultrasonographic techniques into routine HF care offers a promising avenue for improving early congestion detection, personalizing treatment, and ultimately enhancing patient outcomes.

## Aim of work

This exploratory study aimed to investigate the association between the VExUS score and relevant short-term clinical outcomes in ambulatory patients with HFrEF.

### Study design and population

This prospective, observational cohort study was conducted at the heart failure clinic of a tertiary care hospital, which followed primarily patients with heart failure who were discharged from the inpatient department or patients referred from other hospitals. Adult patients (≥ 18 years) with established HFrEF, as defined by European Society of Cardiology (ESC) guidelines, were consecutively enrolled. The exclusion criteria included patients with recent ischemic events (within 3 months) or conditions known to affect VExUS measurements (liver cirrhosis, portal vein thrombosis, inferior vena cava [IVC] thrombus) or inadequate image quality in the ultrasound assessment. The decision to titrate the medications was left for the routine clinical assessment of the treating physician independent of the VExUS score. In this study, the VExUS score was performed by another physician and not by the attending physician in the outpatient clinic. The ethical committee in our hospital approved the study protocol, and all patients provided written informed consent.

### Data collection and measurements

Baseline data collection included demographics, medical history (HF duration, prior hospitalizations, symptom assessment using the New York Heart Association [NYHA] functional classification), cardiovascular risk factors, and medication history (specifically guideline-directed medical therapy [GDMT]. Physical examination included. Laboratory testing comprised hemoglobin, serum electrolytes (sodium, potassium, magnesium, calcium), creatinine, and estimated glomerular filtration rate (eGFR) determined using the Cockcroft–Gault equation. Twelve-lead electrocardiography (ECG) assessed heart rate, rhythm, and conduction abnormalities. Comprehensive transthoracic echocardiography was performed, evaluating left ventricular ejection fraction (LVEF), mitral inflow patterns, right ventricular function, pulmonary artery pressure, tricuspid regurgitation severity, and valvular pathology.

### VExUS score assessment

The VExUS score was assessed using standardized protocols [12]. IVC diameter was measured; Doppler flow patterns in the hepatic, portal, and renal veins were characterized as normal, mildly abnormal, or severely abnormal. The VExUS score was calculated as previously described, ranging from 0 to 3.

### Follow-up and outcomes

Patients were followed for 90 days through monthly visits and phone calls. The primary outcome was a composite end point of all-cause mortality and HF hospitalization within the 90-day follow-up period. Secondary outcomes included unplanned HF visits and worsening renal function (defined as a ≥ 50% increase in serum creatinine from baseline). Hospitalization for heart failure (HF) was characterized as an event that necessitated an overnight stay, along with the presence of HF signs and symptoms and the need for intravenous treatments for HF. Unplanned HF visits involve receiving intravenous diuretics and or increasing the dose of oral diuretics during unscheduled appointments in clinics or emergency departments without hospitalization.

### Statistical analysis

Based on an anticipated event rate of approximately 25% for the composite endpoint of mortality and hospitalization in the low VExUS group with a history of previous hospitalization, we determined the sample size to achieve a statistical power of 80% at a significance level of 0.05. This calculation was performed using standard statistical methods for comparing proportions, which account for the expected differences of 20% in event rates between the two groups. Comparison of categorical variables was performed using the chi-square or Fisher's exact test, as appropriate. Continuous variables were compared using Student's t test for normally distributed data or the Mann–Whitney U test for skewed data. A two-sided *p* value ≤ 0.05 was considered statistically significant. Patients were stratified into two groups based on their VExUS scores (low VExUS: 0–1; high VExUS: 2–3), and outcomes were compared between these groups.

The performance of dilated IVC and high VExUS score in predicting the primary outcome was assessed using sensitivity, specificity, positive predictive value (PPV), and negative predictive value (NPV). These metrics were derived from 2 × 2 contingency tables comparing each predictor (dichotomized IVC and VExUS) to the composite outcome. Version 29 SPSS was used for analysis.

## Results

**Fig. 1:**
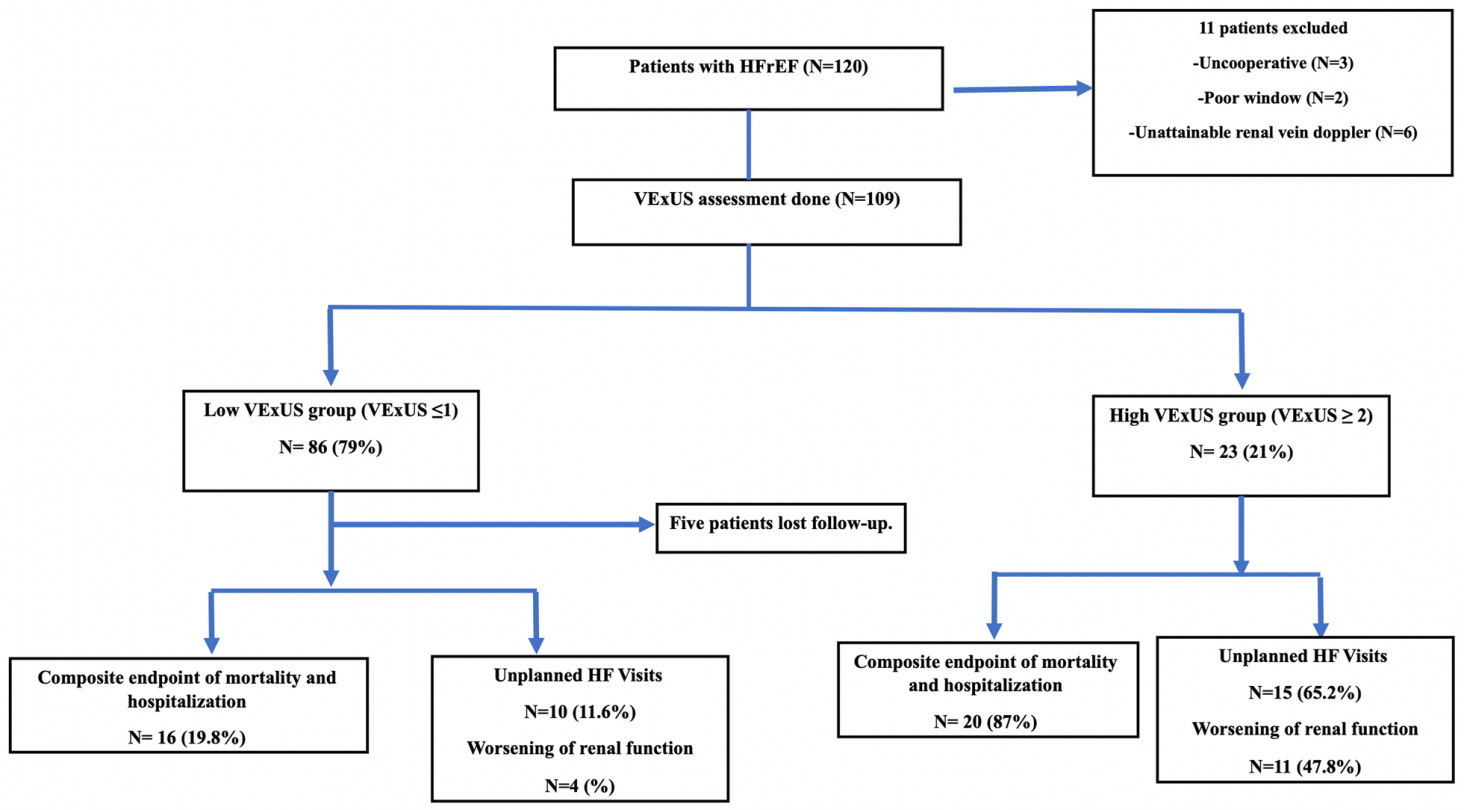
Flow chart of the study population. This flowchart details the patient selection process, from initial screening to final outcome analysis

**Fig. 2:**
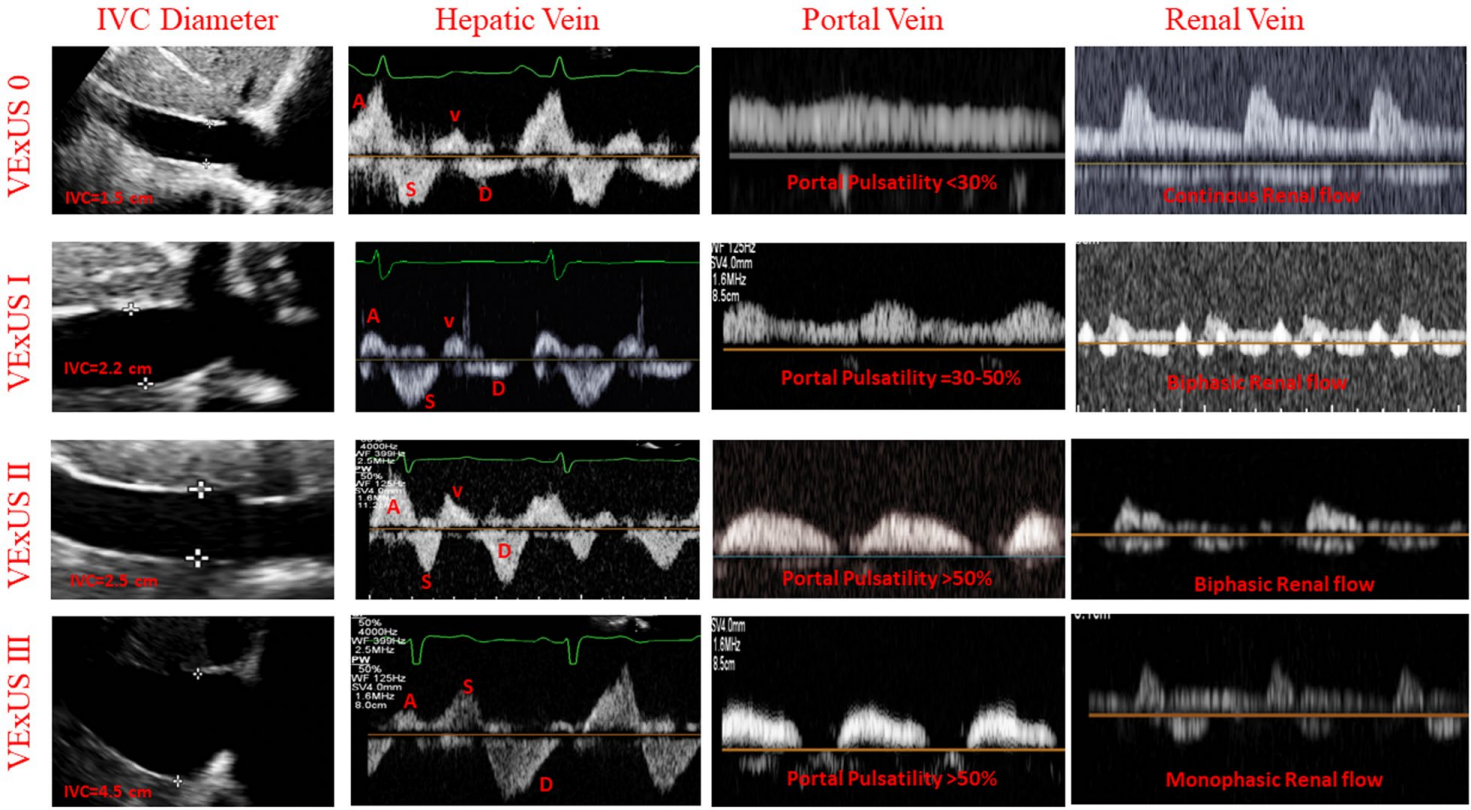
Clinical examples of venous congestion by VExUS score. Representative images demonstrating the ultrasound findings corresponding to different VExUS score grades

Of the 120 patients with HFrEF initially screened, 109 were included in the final analysis due to complete VExUS score assessments. Out of the excluded patients, adequate intrarenal Doppler waveforms were not obtained for 6 of them. The mean additional time required to perform the VExUS assessment beyond the standard echocardiographic study was 5 (± 2) minutes. All 109 patients had a history of previous hospitalization; 20 (18.3%) had an HF hospitalization within six months before enrollment. Of these 109 patients, 86 (79%) had a low VExUS score (0–1), and 23 (21%) had a high VExUS score (≥ 2). Figure 1 depicts the study flowchart, showing that 5 patients were lost to follow-up, leaving 104 for outcome analysis. Figure 2 illustrates examples of different VExUS score grades in four enrolled patients.

### Patient characteristics and comorbidities

The study population was predominantly male. Ambulatory NYHA Class IV functional status was reported in 23 patients (21%). While no statistically significant differences were observed between the high and low VExUS groups in the prevalence of diabetes mellitus, hypertension, chronic kidney disease, or smoking, there was a trend toward a higher prevalence of hypertension (52.2% vs. 30.2%, *p* = 0.05) and CKD (39.1% vs. 19.8%, *p* = 0.053) in the high VExUS group (Table 1). The majority of included patients had HFrEF attributed to ischemic cardiomyopathy, with only a 4.5% proportion having non-ischemic dilated cardiomyopathy.

**Table 1:** Demographic and baseline clinical characteristics of high and low VExUS group

Variable	All (No = 109)	Low VExUS group (No = 86)	High VExUS group (No = 23)	*P* Value
	No. (%)	No. (%)	No. (%)	
Gender, male	89 (81.7%)	68 (79%)	21 (91.3%)	0.178
Age (years)^†^	57 (51–65)	58.5 (52–66)	58 (50–65)	0.52
BMI (kg/m^2^)^†^	27 (23.5–29)	26.2 (23.4–28.6)	27.7 (23.8–29.7)	0.20
SBP (mmHg)^†^	109 (100–120)	110 (100–120)	100 (93–110)	0.014
Heart rate^‡^	83 (± 13)	84 (± 13)	85 (± 17)	0.99
Presence of LL edema	30 (27.5%)	14 (16.3%)	16 (69.6%)	< 0.01
Diabetes	55 (50.5%)	42 (48.8%)	13 (56.5%)	0.51
Hypertension	38 (34.9%)	26 (30.2%)	12 (52.5%)	0.05
CKD	26 (23.9%)	17 (19.8%)	9 (39.1%)	0.053
Duration of HF (months)^†^	12 (8–34.5)	12 (7.6–29.6)	25 (13–36)	0.09
HF Hospitalization in the last 6 months	20 (18.3%)	13 (15.1%)	7 (30.4%)	0.09
Prior coronary intervention				0.60
PCI	65 (60%)	53 (91.6%)	12 (52.2%)	
CABG	6 (5.5%)	4 (4.7%)	2 (8.7%)	
NYHA IV	23 (21%)	12 (14%)	11 (47.8%)	< 0.01
Rhythm				
Sinus	94 (86.2%)	76 (88.4%)	18 (78.3%)	0.20
AF	15 (13.8%)	10 (11.6%)	5 (21.7%)	
Laboratory indices				
Hb level (gm/dl)^†^	11.5 (10–13)	11.5 (10–13)	11.3 (10.5–13)	0.94
Sodium (mg/L)^†^	136 (132–139)	137 (132–139)	136 (130–140)	0.33
Potassium (mg/L)^‡^	4.4 (± 0.6)	4.3 (± 0.6)	4.2 (± 0.7)	0.39
Serum creatinine (mg/L)^†^	1.1 (0.9–1.4)	1.1 (0.9–1.3)	1.3 (1–1.95)	0.05
eGFR (ml/min)^†^	73 (52.8–94.5)	75 (54–98)	67 (45–89)	0.32

*A*F Atrial Fibrillation, *BBB* Bundle Branch Block, *BMI* Body Mass Index, *CKD* Chronic Kidney Disease, *DBP* Diastolic Blood Pressure, *ED* Emergency Department, *eGFR* estimated Glomerular Filtration Rate, *HF* Heart Failure, *IVCD* Intraventricular Conduction Delay, *LBBB* Left Bundle Branch Block, *NYHA* New York Heart Association, *RBBB* Right Bundle Branch Block, *RBBB* Right Bundle Branch Block, *SBP* Systolic Blood Pressure

*eGFR calculated using the Cockcroft–Gault equation

*p*-value of prior coronary intervention (0.60) for both PCI and CABG as one Raw

### Medical therapy among the study population

The high VExUS group had a significantly lower rate of ACE inhibitor/angiotensin receptor blocker (ACEi/ARB) use compared to the low VExUS group (39.1% vs. 66.3%, *P* = 0.015). There were no significant differences between the groups in the use of beta-blockers, mineralocorticoid receptor antagonists (MRA), or sodium-glucose cotransporter-2 inhibitors (SGLT2i). The utilization of angiotensin receptor–neprilysin inhibitor (ARNI) was low among the entire cohort, with no significant difference between both groups (Table 2).

**Table 2:** Baseline medical therapy across the VExUS groups

Medication	All (No = 109)	Low VExUS group (No = 86)	High VExUS group (No = 23)	P Value
Home Diuretic type				
Frusemide	23 (21.1%)	15 (17.4%)	8 (34.8%)	0.19
Torsemide	72 (66%)	59 (68.6%)	13 (56.5%)	
Not on diuretics	14 (12.8%)	12 (14%)	2 (8.7%)	
BB	74 (68%)	61 (71%)	13 (56.5%)	0.189
ACEi/ARBs	66 (60.6%)	57 (66.3%)	9 (39%)	0.015
ARNI	14 (12.8%)	11 (12.8%)	3 (13%)	0.97
MRA	60 (55%)	49 (57%)	11 (47.8%)	0.43
SGLT2	51 (46.8%)	43 (50%)	11 (47.8%)	0.194

*ACEI* Angiotensin-converting Enzyme Inhibitor, *ARBs* Angiotensin Receptor Blockers Inhibitors, *ARNI* Angiotensin Receptor–Neprilysin Inhibitor, *MRA* Mineralocorticoid Receptor Antagonist, *SGLT2* Sodium. Glucose Co-transporter 2 inhibitors, *VExUS* Venous Excess Ultrasound

### Echocardiographic characteristics

In the high VExUS group, patients had significantly lower LVEF (24% vs. 34%, *P* = 0.002), lower TAPSE (16.1 vs. 19.1 mm, *P* < 0.01), lower FAC (30% vs. 37.5%, *P* < 0.01), and higher estimated pulmonary artery systemic pressure (40 vs. 30 mmHg, *P* < 0.01) (Table 3).

**Table 3:** Echocardiographic characteristics of high and low VExUS groups

Variable	All	Low VExUS group	High VExUS group	*P* Value
Assessment of the Left side				
LV Ejection Fraction %	31 (25.5–38)	34 (27–39)	24 (17–35)	0.002
E/A ratio^†^	1.8 (1.1–2.3)	1.5 (1–2.3)	1.85 (1.3–2.3)	0.2
E wave deceleration time (msec)^†^	158 (129–198)	164 (133.5–189)	131 (117–193)	0.195
Severe MR (No, %)	19 (17.4%)	12 (14%)	7 (30.4%)	< 0.01
Assessment of the right side				
TAPSE (mm)^‡^	18.9 (± 4.6)	19.4 (± 4.4)	16.1 (± 4.8)	0.006
FAC of the right ventricle (%)^†^	36 (29–46)	37.5 (30–48)	30 (23–34)	0.005
ePASP(mmHg)^†^	32 (22.5–45)	30.5 (18.2–42.5)	40 (35–58.5)	0.001
TAPSE/PASP ratio^†^	0.6 (0.38–0.83)	0.65 (0.42–0.88)	0.36 (0.27–0.47)	< 0.01
Severe TR (No, %)	13 (11.9%)	5 (5.8%)	8 (34.8%)	< 0.01
TR Vmax(m/sec)^‡^	2.3 (± 0.7)	2.25 (± 0.7)	2.5 (± 0.55)	0.04
RVOT acceleration time (msec)^†^	91 (78.5–115)	101 (80.8–119)	89 (72–99)	0.09
High Echocardiographic probability of pulmonary hypertension	29 (26.6%)	15 (17.4%)	14 (60.9%)	< 0.01

*ePASP* estimated Pulmonary Artery Systemic Pressure, *FAC* Fractional Area Change, *IVC* Inferior Vena Cava, *MR* Mitral Regurgitation, *RV* Right ventricle, *RVOT* Right ventricular acceleration Time, *TR* Tricuspid Regurgitation, *VExUS* Venous Excess Ultrasound Score

†Values expressed as median (25th–75th percentiles)

‡Values expressed as mean (standard deviation)

### The primary and secondary outcomes

**Fig. 3:**
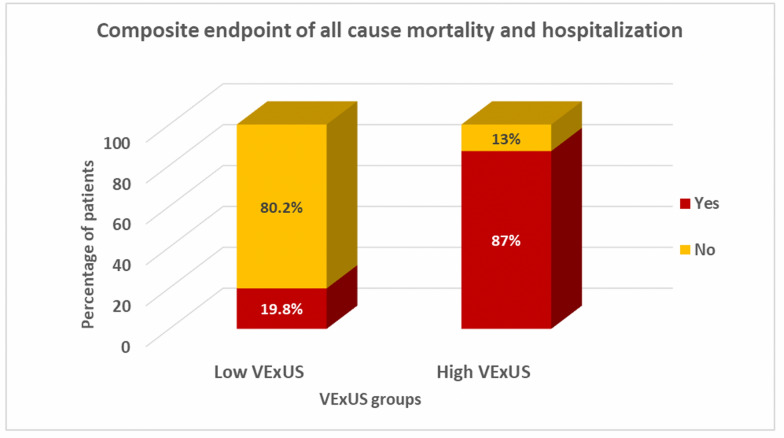
Composite endpoint of all-cause mortality or HF hospitalization among VExUS groups. This bar graph demonstrates a significantly higher rate of the composite endpoint (all-cause mortality or HF hospitalization) in the high VExUS group compared to the low VExUS group

**Fig. 4:**
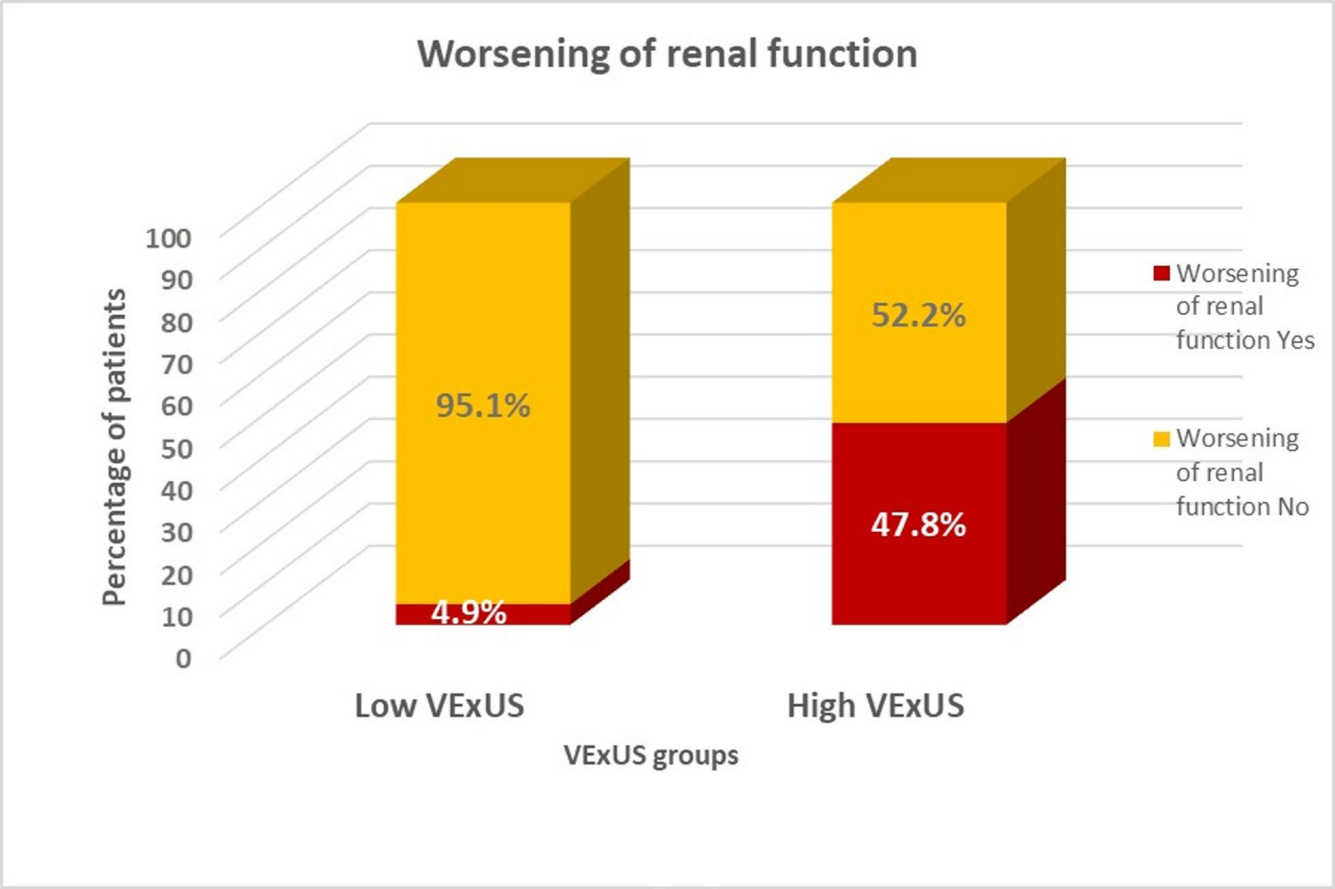
Worsening of renal function among VExUS groups. This bar graph illustrates the significantly higher incidence of worsening renal function in the high VExUS group compared to the low VExUS group

**Fig. 5:**
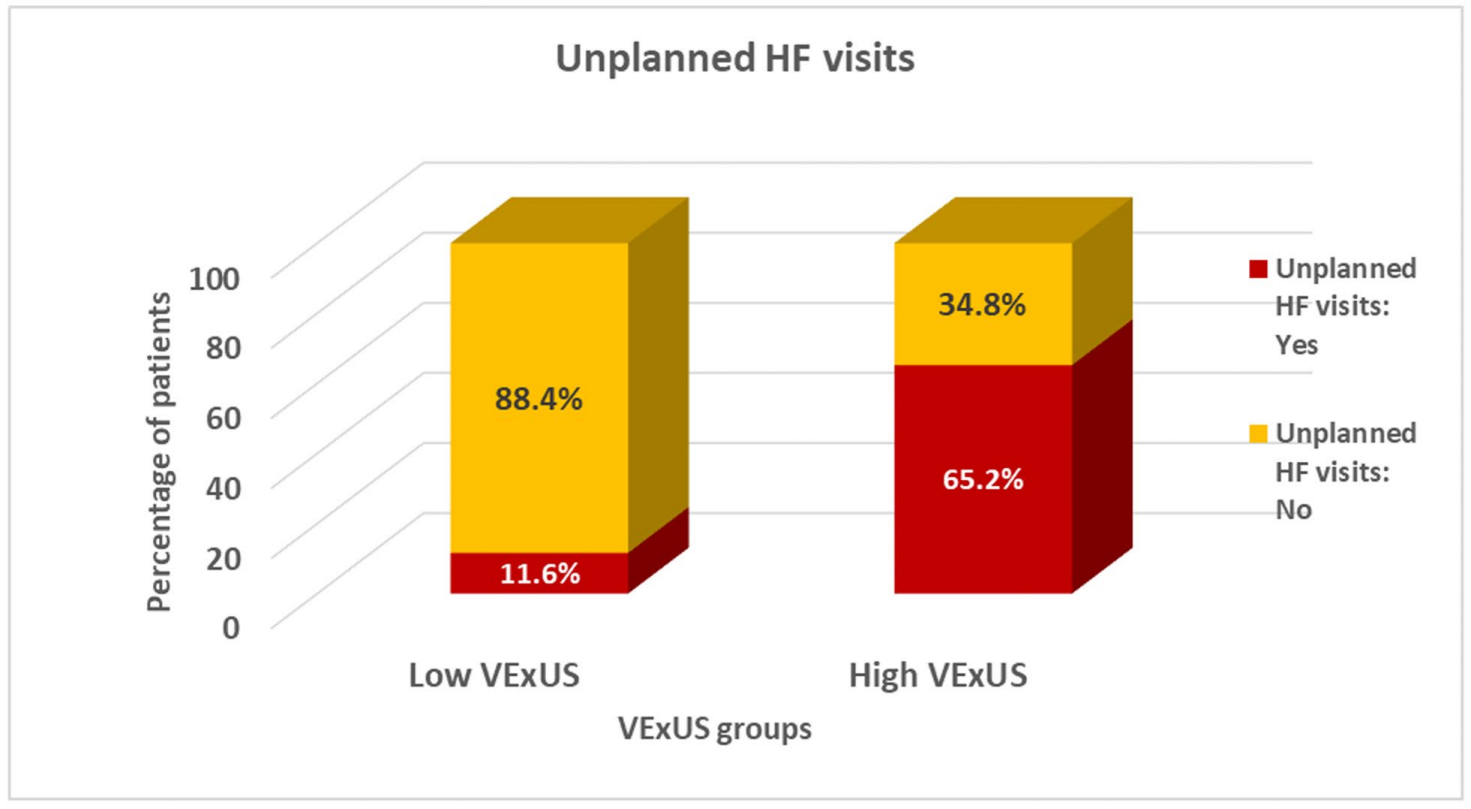
Unplanned HF visits among VExUS groups. This bar graph demonstrates the significantly higher frequency of unplanned HF-related visits in the high VExUS group compared to the low VExUS group

At the end of the three-month follow-up period, the composite endpoint of all-cause mortality, or HF hospitalization, occurred in 36 patients of the 104 patients (34.6%) who completed follow-up. Table 4 summarizes the primary and secondary outcomes stratified by the VExUS group. As shown in Fig. 3, the high VExUS group experienced a significantly higher rate of the primary composite endpoint compared to the low VExUS group (87% vs.19.8%%, *p* < 0.01). Similarly, the high VExUS group demonstrated significantly higher rates of worsening renal function (47.8% vs. 4.9%, *p* < 0.01) (Fig. 4) and unplanned HF-related visits (82.6% vs. 11.6%, *p* < 0.01) (Fig. 5, Table 4).

**Table 4:** Primary and secondary outcomes across VExUS groups

Outcome	All (No. = 104)	Low VExUS group (No. = 81)	High VExUS group (No. = 23)	*P*-Value*
Composite endpoint of mortality and hospitalization No. (%)	36 (34.6%)	16 (19.8%)	20 (87%)	< 0.01
Worsening of renal function,* No. (%)*	15 (14.4%)	4 (4.9%)	11 (47.8%)	< 0.01
Unplanned HF Visits, *No. (%)*	25 (24%)	10 (11.6%)	15 (65.2%)	< 0.01

*VExUS* Venous Excess Ultrasound

A multivariable logistic regression analysis was performed to assess the independent association of a high VExUS score with the composite endpoint of all-cause mortality or HF hospitalization within 90 days, adjusting for age, gender, and the presence of moderate or severe tricuspid regurgitation. A high VExUS score was strongly and independently associated with the composite outcome (OR 22.84, 95% CI 1.77–4.49, *p* < 0.001) (Table 5).

**Table 5:** Association of VExUS Score with a composite of all all-cause Mortality or HF Hospitalization: A Multivariable Logistic Regression Analysis

Predictor	95% Confidence Interval		*p*	Odds ratio
	Lower	Upper		
Age	− 0.0675	0.0397	0.611	0.986
Gender	− 1.3685	1.1934	0.893	0.916
Moderate or Severe TR	− 0.1228	3.2560	0.069	4.790
High VExUS	1.7684	4.4886	<.001	22.840

*TR* Tricuspid Regurgitation; *VExUS* Venous Excess Ultrasound

### Comparison of dilated IVC and high VExUS for predicting

Table 6 presents the performance characteristics of dilated IVC and high VExUS score for predicting the composite outcome. Dilated IVC demonstrated higher sensitivity (86.1%) than a high VExUS score (55.6%). High VExUS exhibited markedly higher specificity (95.9%) than dilated IVC (79.5%). A high VExUS score demonstrated high specificity in this small subgroup (*n* = 13) of moderate to severe tricuspid regurgitation compared to dilated IV (Table 7).

**Table 6:** Comparison of Dilated IVC and High VExUS for Predicting 90-Day all-cause Mortality or HF Hospitalization

Measurement	Outcome (No Event)	Outcome (Event met)	Sensitivity (%)	Specificity (%)	PPV (%)	NPV (%)
Dilated IVC (> 2 cm)	58 (79.5%)	31 (86.1%)	86.1	79.5	67.4	87.7
High VExUS (≥ 2)	70 (95.9%)	20 (55.6%)	55.6	95.9	87.0	76.7

*IVC* Inferior Vena Cava, *VExUS* Venous Excess Ultrasound, *PPV* Positive Predictive Value, *NPV* Negative Predictive Value

**Table 7:** Comparison of Dilated IVC and High VExUS for Predicting 90-day all-cause Mortality or HF Hospitalization in patients with moderate to severe tricuspid regurgitation

Measurement/Score	Outcome = 0 (No Event)	Outcome = 1 (Event)	Sensitivity (%)	Specificity (%)	PPV (%)	NPV (%)
Dilated IVC (> 2 cm)	1 (33.3%)	9 (90%)	90.0	33.3	90.0	33.3
High VExUS (≥ 2)	3 (100%)	8 (80%)	80.0	100	72.7	100

*IVC* Inferior Vena Cava, *VExUS* Venous Excess Ultrasound, *PPV* Positive Predictive Value, *NPV* Negative Predictive Value

## Discussion

This study investigated the utility of the VExUS score in ambulatory patients with HFrEF, focusing on its potential association with short-term adverse outcomes in this population.

Traditional assessment of heart failure severity and prognosis has relied heavily on clinical symptoms and, more recently, biomarkers such as B-type natriuretic peptide (BNP) [18]. However, the sensitivity of this assessment in early or less acute settings may not fully capture the extent of venous congestion [19, 20]. The VExUS score, incorporating multiple ultrasonographic parameters, offers a comprehensive assessment of venous congestion, making it particularly useful in ambulatory settings [11].

## Key findings

Our study demonstrates a significant association between a VExUS ≥ 2 score and increased adverse cardiovascular events. A high VExUS score was associated with significantly higher rates of composite all-cause mortality and HF hospitalization, worsening renal function, and unplanned HF visits.

These findings align with the established understanding that venous congestion is a crucial factor driving heart failure progression and adverse outcomes [21, 22]. These results build upon previous studies that have examined the VExUS score in acute heart failure populations [8, 12, 23–25].

### Comparison with the existing literature

The cohort with ambulatory heart failure in our study explains the lower prevalence of high VExUS grades (21%, *N* = 23) compared to previous studies. Consistent with previous studies [8, 12, 25], our findings demonstrate a clear association between high VExUS scores and indicators of right ventricular dysfunction, including reduced TAPSE and FAC, the presence of severe tricuspid regurgitation, and elevated estimated pulmonary artery systolic pressure. These findings further support the concept that elevated VExUS scores reflect maladaptation of the RV to compensate for increased preload, a hallmark of venous congestion and potentially contributing to subsequent adverse clinical outcomes [8, 12, 23–25].

While the previous studies have focused on the VExUS 3 category as an indicator of severe congestion and its correlation with adverse outcomes, our study, focusing on a distinct cohort of ambulatory patients, utilized a combined VExUS 2 and 3 category to represent a high VExUS score. This approach, driven by the lower prevalence of VExUS 3 scores in our ambulatory population, allowed for a clinically relevant comparison between high and low VExUS groups.

Patients with high VExUS grades (VExUS ≥ 2) experienced significantly worse clinical outcomes over the three-month follow-up period compared to those with low VExUS grades (VExUS ≤ 1). The high VExUS group demonstrated a significantly higher rate of the primary composite endpoint (mortality or HF hospitalization) compared to the low VExUS group. Furthermore, the high VExUS group experienced substantially higher rates of worsening renal function and unplanned HF-related healthcare utilization.

Our findings on the VExUS's prognostic utility in HF align with prior studies on VExUS's prognostic utility in HF, although previous studies focused primarily on acute or critically ill patients. Landi et al. [23] found that VExUS 3 predicted mortality and readmission in acutely decompensated HF. Rinaldi et al. [24], using a modified VExUS, also showed its ability to predict HF-related readmissions or emergency visits. Anastasiou et al. [12] observed that VExUS 3 correlated with higher in-hospital mortality, increased inotrope use, and higher furosemide doses in critically ill acute HF, further supporting the link between congestion and adverse outcomes. Our study extends these observations to the ambulatory setting, suggesting that VExUS may identify patients at risk before acute decompensation, offering an opportunity for earlier intervention.

### The discriminative ability of VExUS scores versus IVC diameter assessment alone

Dilated IVC showed numerically higher sensitivity than a high VExUS score, suggesting it may be more effective at detecting patients at risk for the composite endpoint. However, high VExUS exhibited markedly higher specificity than dilated IVC, implying a lower rate of false positives. In the ambulatory setting of this study, where the prevalence of severe congestion might be lower than in hospitalized cohorts, the higher specificity of VExUS could potentially reduce unnecessary medical interventions or hospitalization. This difference may be due to the VExUS score's more comprehensive assessment of venous congestion, incorporating additional parameters beyond IVC diameter, while IVC is still a good sensitive tool.

Our analysis revealed a significant interaction between tricuspid regurgitation and the performance of both dilated IVC and high VExUS score in predicting the composite endpoint. While dilated IVC demonstrated a sensitive tool to predict venous congestion and subsequent clinical events, its specificity was markedly low in the presence of moderate or severe TR. This may be due to the direct influence of TR on IVC diameter, making it a less specific indicator of systemic venous congestion in this subgroup.

Conversely, the full VExUS score, incorporating additional hemodynamic parameters, showed better discriminatory ability even in patients with moderate or severe tricuspid regurgitation. The high specificity of VExUS in this subgroup is particularly noteworthy, as it indicates a very low rate of false positives; however, the small sample size, particularly in the subgroups stratified by TR, limits the ability to make a definitive conclusion. These findings highlight the potential importance of considering TR severity when interpreting IVC and VExUS assessments and raise the possibility that VExUS offers a significant advantage over IVC assessment alone in ambulatory HFrEF patients with moderate or severe TR.

Finally, it is crucial to emphasize the dual nature of systemic venous congestion, as reflected by VExUS or IVC parameters, particularly within the context of HFrEF. While elevated venous pressures are a hallmark consequence of right ventricular dysfunction and a component of right-sided heart failure, the pathophysiology in HFrEF often involves significant contributions from neurohormonal activation and cardiorenal interaction even beyond the effect of associated RV dysfunction alone. These mechanisms drive systemic sodium and water retention, leading to a state of true hypervolemia that contributes significantly to venous congestion, sometimes preceding or occurring alongside progressive worsening of the overall heart failure syndrome. Understanding this complex interplay underscores why assessing the degree of congestion, as achieved with VExUS in our study, provides potential prognostic information regarding the risk of all HF-related events [26, 27]^.^ An important practical consideration for the clinical implementation of VExUS is its technical feasibility. In our study, conducted in an outpatient heart failure clinic setting, we achieved a high success rate (> 90%) in obtaining adequate images for complete VExUS scoring, suggesting good feasibility in this specific ambulatory population. This agrees with experiences reported in critical care settings and previous studies [28], although challenges can vary by clinical environment and patient factors. While VExUS requires training, our findings support its potential applicability beyond the intensive care unit, particularly in specialized heart failure clinics. However, acknowledging the technical demands, particularly associated with obtaining optimal renal vein Doppler signals, the potential utility of simplified VExUS protocols warrants exploration. For instance, omitting the renal vein assessment could enhance feasibility, reduce examination time, and lower the training barrier; however, it needs further exploration.

## Limitations

Our study has some limitations that warrant consideration. First, as a single-center study, the findings may not be generalizable to the broader population. Second, the observational design precludes definitive conclusions about causality. Third, the short 90-day follow-up duration limits the assessment of long-term prognostic value, although it could serve with our specific focus on short-term risk. Therefore, we emphasize the study's exploratory goals in this context. Fourth, our sample size calculation, which was based on data from studies including recently discharged HFrEF patients, may have overestimated the event rate anticipated in our ambulatory cohort. While those studies informed our initial power analysis, our cohort had a lower-than-expected prevalence of very recent HF hospitalization. Further, this reinforces the exploratory nature of this study and the need for larger long-term studies. Fifth, inherent potential variability in ultrasound measurements exists, although standardized protocols were utilized to minimize this.

## Conclusions

This study demonstrates that elevated VExUS scores are associated with adverse short-term outcomes in ambulatory HFrEF patients. VExUS score offers a more comprehensive assessment of venous congestion than IVC diameter alone and may be particularly valuable in patients with significant TR. Larger, prospective multicenter studies are warranted to confirm these findings and establish the optimal role of VExUS in managing ambulatory HFrEF.

## Data Availability

The data supporting the results of this research are available upon request.

## Abbreviations

ADHF: Acute Decompensated Heart Failure
AF: Atrial Fibrillation
AKI: Acute Kidney Injury
ARB: Angiotensin Receptor Blocker
ARNI: Angiotensin Receptor–Neprilysin Inhibitor
AUC: Area Under the Curve
BB: Beta-blocker
BMI: Body Mass Index
BNP: B-type Natriuretic Peptide
CABG: Coronary Artery Bypass Graft
CKD: Chronic Kidney Disease
ECG: Electrocardiography
ED: Emergency Department
eGFR: Estimated Glomerular Filtration Rate
ESC: European Society of Cardiology
FAC: Fractional Area Change
GDMT: Guideline-Directed Medical Therapy
HF: Heart Failure
HFrEF: Heart Failure with reduced Ejection Fraction
IVC: Inferior Vena Cava
LVEF: Left Ventricular Ejection Fraction
MR: Mitral Regurgitation
MRA: Mineralocorticoid Receptor Antagonist
NPV: Negative Predictive Value
NYHA: New York Heart Association
PCI: Percutaneous Coronary Intervention
PPV: Positive Predictive Value
RAP: Right Atrial Pressure
ROC: Receiver Operating Characteristic
RV: Right Ventricle
RVOT: Right Ventricular Outflow Tract
SGLT2i: Sodium-Glucose Cotransporter-2 Inhibitor
SPSS: Statistical Package for the Social Sciences
TAPSE: Tricuspid Annular Plane Systolic Excursion
TR: Tricuspid Regurgitation
VExUS: Venous Excess Ultrasound
